# Targeting Driver Oncogenes and Other Public Neoantigens Using T Cell Receptor–Based Cellular Therapy

**DOI:** 10.1146/annurev-cancerbio-061521-082114

**Published:** 2023-01-25

**Authors:** Tijana Martinov, Philip D. Greenberg

**Affiliations:** 1 Program in Immunology and Clinical Research Division, Fred Hutchinson Cancer Center, Seattle, Washington, USA; 2 Immunology Department, University of Washington, Seattle, Washington, USA

**Keywords:** immunotherapy, adoptive cellular therapy, neoantigen, T cell receptor

## Abstract

T cell reactivity to tumor-specific neoantigens can drive endogenous and therapeutically induced antitumor immunity. However, most tumor-specific neoantigens are unique to each patient (private) and targeting them requires personalized therapy. A smaller subset of neoantigens includes epitopes that span recurrent mutation hotspots, translocations, or gene fusions in oncogenic drivers and tumor suppressors, as well as epitopes that arise from viral oncogenic proteins. Such antigens are likely to be shared across patients (public), uniformly expressed within a tumor, and required for cancer cell survival and fitness. Although a limited number of these public neoantigens are naturally immunogenic, recent studies affirm their clinical utility. In this review, we highlight efforts to target mutant KRAS, mutant p53, and epitopes derived from oncogenic viruses using T cells engineered with off-the-shelf T cell receptors. We also discuss the challenges and strategies to achieving more effective T cell therapies, particularly in the context of solid tumors.

## INTRODUCTION

The immune system has evolved to identify, respond to, and eliminate threats to homeostasis. Cells of the adaptive immune system can distinguish foreign from self substances by engaging their antigen receptors. T cell receptors (TCRs) recognize peptide fragments derived from intracellular or endocytosed proteins presented in the context of a groove in major histocompatibility complexes (MHCs) (Hogquist & Jameson 2014). T cells that react too strongly to self-peptide:MHC are pruned from the repertoire during thymic development or silenced in the periphery through a variety of mechanisms in attempts to thwart autoimmunity (Hogquist & Jameson 2014). T cells with an acceptably low affinity for self persist in the periphery, where they survey antigen-presenting cells (APCs) in search for higher-affinity interactions with their cognate antigen that can trigger activation. As TCRs are generated through a random recombination process, and each developing T cell expresses a unique receptor, the overall T cell repertoire is highly diverse and can mount immune responses to a multitude of foreign antigens (Robins et al. 2010).

Cancer arises from normal cells due to spontaneous or environmentally induced genetic alterations that collectively lead to genomic instability, loss of proliferative control, and resistance to cell death (Hanahan & Weinberg 2011). Genomic and epigenetic changes can increase cancer cell fitness, but this comes at a cost—the more the cancer cell proteome diverges from normal cells, the greater the likelihood it will elicit an immune response (Schumacher & Schreiber 2015). After decades of debate, it is now widely accepted that immune cells interact with cancer cells and influence cancer evolution through a process known as cancer immunosurveillance/editing (Schreiber et al. 2011, Ward et al. 2016). Specifically, immune cell–cancer cell interactions can result in cancer elimination, in an equilibrium state in which tumor outgrowth is contained, or in cancer escape with tumor progression (Chen & Mellman 2013, Schreiber et al. 2011, Ward et al. 2016). Cancer elimination involves successful priming of tumor antigen–specific T cells in tumor-draining lymph nodes by dendritic cells that have processed tumor-derived antigens, subsequent T cell trafficking to the tumor, and effective T cell–mediated tumor destruction. If any tumor cells evade T cell surveillance at this stage, equilibrium can ensue, with tumor containment and continuous immune-mediated evolutionary pressure. In some cases, equilibrium might persist for the lifetime of the host, while in other cases, cancer cells can escape T cell control and grow into clinically apparent tumors. Cancer escape can occur through a variety of mechanisms, including loss of rejection antigen(s), establishment of an immunosuppressive tumor microenvironment (TME), and progressive exhaustion or deletion of reactive T cells. The goal of cancer immunotherapy is to reinvigorate existing antitumor immunity, such as by immune checkpoint blockade (ICB), actively inducing and stimulating de novo antitumor responses (vaccination), or providing ex vivo manipulated tumor-specific T cells [adoptive T cell therapy (ACT)] to achieve tumor clearance. ICB aims to restore or establish functional endogenous tumor-reactive T cells by preventing inhibitory receptor signaling. ICB has led to remarkable clinical responses in melanoma (~30%), non-small-cell lung cancer (~45%), and classic Hodgkin’s lymphoma (60–85%), among others, but most cancer patients do not benefit from ICB (Ribas & Wolchok 2018). Resistance to ICB is largely due to either the paucity of endogenous T cells with a high affinity for tumor-specific antigens (TSAs) or profound and irreversible T cell dysfunction (Kalbasi & Ribas 2020). Therefore, strategies that induce or provide an adequate de novo immune response to tumor antigens are urgently needed.

Identifying and harnessing cancer antigens that drive effective immune recognition are critical for the success of immunotherapy. Cancer rejection antigens described to date can be broadly divided into tumor-associated antigens (TAAs) and TSAs (Pearlman et al. 2021, Verdon & Jenkins 2021). TAAs include peptide epitopes derived from self-proteins that are (*a*) overexpressed in cancer cells compared to normal tissues, (*b*) expressed in cancer cells and in the tissue of origin during tissue development, or (*c*) expressed in cancer cells and in germ cells of the testes/ovaries. TSAs, also known as neoantigens, include peptide products of (*a*) nonsynonymous mutations, (*b*) insertion/deletion events, (*c*) altered splicing, (*d*) transcription and translation of noncoding regions of DNA, (*e*) translocations, or (*f*) aberrant posttranslational modifications (Coulie et al. 1995, Jayasinghe et al. 2018, Laumont et al. 2018, Lennerz et al. 2005). In virally induced malignancies, TSAs also include peptides derived from viral oncogenes (Pearlman et al. 2021, Verdon & Jenkins 2021, Ward et al. 2016).

While TSA-specific T cells were long speculated to contribute to naturally occurring or therapeutically induced immunity, this hypothesis proved difficult to test, largely due to technological limitations. The development of next-generation sequencing platforms and algorithms that predict epitope processing, MHC binding, and presentation enabled deeper dives into the cancer genome and immunopeptidome (Nielsen et al. 2003, Rammensee et al. 1999, Vita et al. 2015). Recent studies have shown that neoantigen-specific T cells are detectable in tumor biopsies, expand following ICB, and directly mediate tumor regression (Leidner et al. 2022; Parkhurst et al. 2019; Stevanovic et al. 2017; Tran et al. 2016, 2014; van Rooij et al. 2013; Zacharakis et al. 2018). Neoantigen burden, immunogenicity, and clonality influence response to ICB, as demonstrated across multiple patient cohorts and tumor indications (Goodman et al. 2020; McGranahan et al. 2015, 2016; Riaz et al. 2017; Rizvi et al. 2015; Snyder et al. 2014). Collectively, the evidence suggests boosting or introducing T cell reactivity to tumor neoantigens is beneficial and highly desirable.

Unlike prophylactic vaccines against pathogens, cancer vaccines have yielded limited clinical benefit to date and require further optimization before becoming reproducibly effective (Hollingsworth & Jansen 2019). This is largely because of limited knowledge of (*a*) how to best construct and deliver an immunogenic therapeutic vaccine for an antigen already present in the host, (*b*) how to overcome systemic immune dysregulation in cancer patients, and (*c*) how to induce cell responses that can surmount the immunosuppressive TME (Hollingsworth & Jansen 2019). ACT is a promising alternative approach that involves the transfer of large numbers of ex vivo–manipulated tumor antigen–specific T cells. ACT modalities include the administration of expanded tumor-infiltrating lymphocytes (TILs) or tumor-reactive T cells from peripheral blood, and the infusion of T cells engineered to express either a tumor-specific chimeric antigen receptor (CAR) or a tumor-specific TCR. Although proof-of-concept ACT studies were performed with ex vivo-expanded TILs, this approach, which depends on the quality and quantity of infiltrating T cells, has been successful only in a limited number of indications, all of which require significant T cell infiltration into the tumor (Chandran & Klebanoff 2019, Leko & Rosenberg 2020). T cell engineering provides an opportunity to overcome these limitations by redirecting the specificity of large numbers of T cells and by rewiring T cell signaling and metabolism for improved fitness in the TME. CAR- and TCR-engineered T cells differ with respect to their antigen recognition domain, sensitivity, and downstream signaling (Chandran & Klebanoff 2019). CARs incorporate antibody-based ligand-binding domains, which can recognize proteins on the cancer cell surface and transmit a signal through a synthetic CD3ζ-CD28 or CD3ζ-4-1BB intracellular domain. TCRs recognize peptides derived from surface and intracellular proteins loaded into HLA/MHC class I or II molecules, and signal using T cell–intrinsic machinery. Most CAR and TCR T cells used to date target TAAs and have led to robust responses in some patients (Chandran & Klebanoff 2019, Leko & Rosenberg 2020). However, on-target off-tumor activity is an issue, particularly with respect to CAR T cells, as finding a surface protein exclusively expressed on tumor cells has been a challenge. As TCRs can respond to peptides from intracellular tumor-specific neoantigens, there is a rationale to develop neoantigen-specific TCR-based therapies.

Most neoantigens arise from stochastic genetic/epigenetic aberrations occurring throughout tumor evolution from poor replication fidelity, are variably expressed by cells within a tumor, and do not contribute to the malignant phenotype (Schumacher & Schreiber 2015). Thus, most neoantigens are unique to each patient and are considered private. Developing personalized neoantigen vaccines or T cell products for such antigens is feasible in principle, but challenging from a manufacturing and regulatory perspective, as each product is tailored and only applicable to a single patient (Carreno et al. 2015, Jacoby et al. 2019, Kotsiou et al. 2020, Ott et al. 2017, Sahin et al. 2017). Furthermore, it can take weeks or months from the initial biopsy to identify candidate neoantigens until the formulation of a customized deliverable product. This treatment delay could enable tumor progression or immunoediting and, thus, diminish treatment efficacy.

A smaller subset of neoantigens includes epitopes that span recurrent mutation hotspots, translocations, or gene fusions in oncogenic drivers or tumor suppressors, as well as epitopes that arise from viral oncogenic proteins and that are characteristically found in particular tumors. These neoantigens are more likely to be clonal (expressed in the tumor evolutionary trunk) and shared across different patients (i.e., public neoantigens), as their source proteins are required for cancer cell survival and fitness (Makohon-Moore et al. 2017, Schumacher & Schreiber 2015). To date, over 2,000 cancer driver mutations have been identified, although only ~20 have been characterized as immunogenic and actionable (Table 1) (Bailey et al. 2018, Castle et al. 2019, Pearlman et al. 2021). Factors that influence neoantigen immunogenicity include (*a*) source protein expression levels, (*b*) source protein processing, (*c*) peptide:MHC binding, (*d*) surface peptide:MHC stability, and (*e*) T cell–mediated recognition (Luksza et al. 2017). In this review, we highlight some of the efforts to target public neoantigens using TCR-based T cell therapy, using mutant KRAS (mKRAS), mutant p53, and viral oncogenes as case studies. We also discuss the challenges that must be overcome to achieve more broadly applicable and reproducibly effective T cell therapies, particularly in the context of solid tumors.

## MUTANT KRAS–DIRECTED T CELL THERAPY

### Mutant KRAS Is a Common Oncogenic Driver

Genes in the RAS family (*HRAS, KRAS, NRAS*) represent the most frequently mutated oncogenes in human cancers, accounting for 3.4 million cancer diagnoses each year (Prior et al. 2020). In normal cells, RAS is localized at the cell membrane and functions as a GTPase. During quiescence, RAS is inactive and bound to GDP. Growth factor receptor signaling recruits guanine nucleotide exchange factors that catalyze GTP loading and RAS activation. GTP-bound RAS directly activates the MAPK (RAF/MEK/ERK) and the PI3K/AKT pathways, leading to cell growth and proliferation. Active RAS is tightly regulated, with GTPases promptly returning active RAS to its inactive GDP-bound state (Moore et al. 2020, Ostrem & Shokat 2022).

Mutations in *KRAS* represent ~75% of all RAS mutations and commonly occur at residues G12, G13, and Q61, which interface with the nucleotide-binding pocket, leading to impaired GTP hydrolysis or enhanced GDP exchange for GTP (Moore et al. 2020). mKRAS is a potent tumor driver, as it can activate downstream growth and proliferation signaling in the absence of extracellular cues. KRAS G12 mutations are found in ~30% of colorectal cancers, ~30% of lung adenocarcinomas, and ~80% of pancreatic ductal adenocarcinomas, with G12C, G12V, and G12D substitutions being the most common (Moore et al. 2020).

Over the last 40 years, many groups have attempted to develop selective mKRAS inhibitors. Among the G12 mutant variants, KRAS G12C still retains some GTP hydrolytic activity and can cycle between GTP- and GDP-bound states. Shokat and colleagues developed compounds (reviewed in Ostrem & Shokat 2022) that form a disulfide bond with the G12C residue and covalently trap KRAS in a GDP-bound state. These findings prompted the development and testing of additional KRAS G12C inhibitors AMG510 (sotorasib) and MRTX849 (adagrasib). While both drugs have led to exciting results with disease control in 37–53% of patients, development of resistance has been common and well documented, and includes KRAS G12C amplification, secondsite KRAS mutations that inhibit drug binding/activity, and downstream activating mutations in theMAPK and PI3K pathways (Ostrem & Shokat 2022). To circumvent resistance, phase I/II trials are combining KRAS G12C inhibitors with MAPK or PI3K pathway inhibitors, or with ICB. Although KRAS G12C is the most common KRAS mutation in lung cancer, G12V and G12D driver mutations predominate in pancreatic cancers. Efforts to drug KRAS G12V and G12D have proven more difficult, as there is no reactive cysteine in the GTP/GDP binding site to facilitate covalent interactions. A recent structure-based approach to drug design yielded a promising noncovalent KRAS G12D inhibitor (MRTX1133). MRTX1133 interacts with active and inactive KRAS G12D forms, and significantly reduces the growth of KRAS G12D–mutant tumor cell lines and patient-derived xenografts (Hallin et al. 2022). Additional strategies to target KRAS G12D and G12V are being pursued, including interfering with KRAS posttranslational processing, targeting downstream signaling effectors, and harnessing the immune response (see below).

### Mutant KRAS Is Immunogenic

Studies in mice in the early 1990s demonstrated that oncogenic mKras is immunogenic, as vaccination with mKras peptides resulted in expansion of CD4^+^ and CD8^+^ T cells that specifically recognized mutant, but not wild-type Kras protein. Additionally, expanded CD8^+^ T cells killed mKras-expressing target cells in vitro and in vivo, demonstrating mKras-derived mutation-containing peptides were processed and presented (Cheever et al. 1993, Peace et al. 1991). This prompted the development and testing of peptide-based human mKRAS vaccine strategies (Gjertsen et al. 1995, Gjertsen et al. 2001), with a suggestion from one study that patients who responded to vaccination had a longer overall survival compared to patients who did not respond (Gjertsen et al. 2001). Subsequently, nanoparticles, mRNA-based vaccines, and heat-killed yeast (GI-4000) were examined as vaccine vectors, but with limited success (Chaft et al. 2014, Muscarella et al. 2021).

### Developing Adoptive T Cell Therapy Targeting Mutant KRAS

Direct evidence for both mKRAS immunogenicity and the utility of providing mKRAS-specific CD8^+^ T cells came from studies at the National Cancer Institute (NCI). Tran et al. (2016) cocultured TILs from resected metastatic colorectal carcinoma lesions with autologous APCs transduced to express the patient’s sequenced tumor neoantigens. One TIL culture, which showed a response to KRAS G12D, was expanded for treatment. At the time of infusion, ~75% of CD8^+^ T cells in the cell product were specific for the KRAS G12D epitope and produced multiple effector cytokines. Adoptive transfer of these cells led to regression of seven lung lesions, and a 9-month-long response. TCR sequencing of the infusion product revealed four clonally expanded TCRs that recognized KRAS G12D in the context of the HLA-C*08:02 allele and were functional in transduced CD8^+^ T cells from unrelated healthy donors. A recent case report detailed the use of two of these TCRs in a patient with metastatic pancreatic cancer whose tumor had become refractory to standard care and did not respond to prior TIL therapy (Leidner et al. 2022). For that patient, autologous peripheral blood CD4^+^ and CD8^+^ T cells were retrovirally transduced to express TCRs specific for KRAS G12D:HLA-C*08:02 complexes. Prior to infusion, 90% of the cells expressed mKRAS TCRs. The patient was preconditioned with tocilizumab and cyclophosamide and given IL-2 starting at 18 hours after infusion, for a total of five doses. At 1 month postinfusion, shrinkage of lung lesions was evident, and at 6 months this response remained ongoing with detectable TCR-transduced T cells in blood, albeit at lower levels than before. Another pancreatic cancer patient was treated using the same TCRs, for whom the transduction efficiency was lower (67% of cells expressed the TCRs) and the transduced T cells appeared more differentiated. The patient did not receive tocilizumab and developed grade 3 cytokine release syndrome and grade 2 immune-related neurotoxicity. At 1 month, modest shrinkage of liver lesions was observed, but the response was transient, and the patient succumbed to disease 6 months after treatment. The authors are still investigating the mechanisms of treatment failure and toxicity. While more work remains to be done, this proof-of-principle study suggests targeting mKRAS with TCR-based T cell therapies is feasible and can lead to tumor control.

HLA-C*08:02 is projected to be expressed in ~7% of the total US population, and KRAS G12D–mutant cancers are estimated to arise in at least 4,000 HLA-C*08:02^+^ patients annually (Gonzalez-Galarza et al. 2021, Pearlman et al. 2021). To expand mKRAS-specific TCR-targeted therapies, researchers have isolated TCRs restricted to other HLA class I alleles. Wang et al. (2016) immunized HLA-A*11:01-transgenic mice with KRAS G12V and KRAS G12D epitopes predicted to bind to HLA-A*11:01, isolated and propagated responding CD8^+^ T cells, and sequenced the TCRs. TCR specificity and function were validated in transduced CD8^+^ T cells from healthy human donors. KRAS G12V- and G12D-specific TCRs recognized peptide-pulsed APCs and tumor cells. Furthermore, adoptive therapy with TCR-transduced T cells targeting G12D prolonged survival in a xenograft model. These TCRs are currently being evaluated in clinical trials (http://clinicaltrials.gov/ identifiers NCT03190941 and NCT03745326), but it is important to note that the TCRs are of murine origin. To avoid the associated risk of host-mediated rejection of the cell therapy product, we and other groups have isolated fully human mKRAS-specific TCRs restricted to HLA-A*11:01 and other common alleles for broader clinical use (Bear et al. 2021, Cafri et al. 2019, Choi et al. 2021, Martinov et al. 2022).

Bear et al. (2021) used epitope prediction algorithms to identify mKRAS peptides encompassing G12 mutations (G12V, G12D, G12R, and G12C) likely to bind to HLA-A*02:01, HLA-A*03:01, HLA-A*11:01, and HLA-B*07:02 alleles. Epitopes with predicted affinity for HLA less than 500 nM were experimentally validated in HLA competitive binding assays, and natural processing of these epitopes was assessed by targeted mass spectrometry of HLA-bound peptides in cell lines that expressed a single defined HLA allele and an mKRAS minigene. These studies revealed that KRAS G12V, G12D, and G12R epitopes can be eluted from HLA-A*03:01, HLA-A*11:01, and HLA-B*07:02. CD8^+^ T cells from healthy donors were cultured with autologous APCs pulsed with the identified mKRAS peptides, and the responding cells were sorted with tetramers for TCR sequencing. Three TCRs targeting KRAS G12V:HLA-A*03:01, KRAS G12V:HLA-A*11:01, and KRAS G12R:HLA-B*07:02 were selected for further study. All three TCRs recognized endogenously processed and presented peptides in vitro. T cells expressing the G12V-specific TCRs were also able to control tumor growth in 60–80% of NSG (NOD scid gamma) mice in vivo in a tumor xenograft model, affirming the potential therapeutic activity of utilizing these TCRs.

Choi et al. (2021) used high-resolution mass spectrometry to characterize the HLA-bound peptides isolated from cells engineered to concurrently express mKRAS minigenes (encompassing the first 35 amino acids from G12V, G12D, G12R, and G12C variants) and individual HLA class I alleles (HLA-A*03:01, HLA-A*11:01, HLA-A*30:01, HLA-A*68:01, HLA-B*07:02, HLA-C*01:02, HLA-C*03:03, HLA-C*03:04, or HLA-C*08:02). In addition to isolating previously validated mKRAS:HLA pairs (Bear et al. 2021, Tran et al. 2016, Wang et al. 2016), 20 other actionable mKRAS:HLA epitopes were detected, and all but one epitope elicited T cell responses from healthy donors. mKRAS-specific T cells restricted to HLA-A*03:01 and HLA-A*11:01 were sorted and sequenced because these alleles are very common in the United States (~20% and 10% of the population, respectively) and at least ~21,000 newly diagnosed patients/year have KRAS G12V/G12D cancers that express either allele (Pearlman et al. 2021). The specificity of the TCRs was validated with in vitro killing assays, but similar to the studies by Bear et al., TCR safety was not evaluated. By virtue of recognizing a tumor neoantigen, mKRAS-specific TCRs are already significantly derisked compared to TCRs targeting TAAs. However, TCRs do have the potential to recognize alternative peptide:HLA complexes, and in some ACT trials, this has led to severe toxicity and fatal outcomes (Cameron et al. 2013, Morgan et al. 2013). Additionally, TCRs harbor an inherent affinity for HLA molecules, which can lead to alloreactivity. Thus, prior to advancing any of these candidates into the clinic, it will be important to ensure by testing that they exhibit neither cross-reactivity nor alloreactivity.

Over 40% of the US population carries the HLA-A*02:01 allele, but efforts to isolate HLA-A*02:01-restricted mKRAS TCRs have proven more difficult. KRAS G12V_5–14_ has a favorable epitope prediction score for binding to HLA-A*02:01 (<500 nM), but attempts to elute this epitope from the HLA-A*02:01 peptide binding groove have yielded mixed results (Bear et al. 2021, Choi et al. 2021, Douglass et al. 2021, Rive et al. 2020). CD8^+^ T cells that recognize KRAS G12V_5–14_ have been isolated from peripheral blood of HLA-A*02:01^+^ healthy donors and patients with pancreatic cancer and shown to kill tumor cells, albeit at very high effector to target ratios (Kubuschok et al. 2006, Rive et al. 2020). The need for high effector to target ratios suggests that fewer KRAS G12V_5–14_:HLA-A*02:01 complexes may be expressed on the cell surface compared to other KRAS peptide:HLA complexes, which are estimated at 8 to 70 copies per cell (Bear et al. 2021, Choi et al. 2021). It has been suggested that mKRAS could also undergo aberrant proteasomal processing, yielding a spliced KRAS G12V_5–6/8–14_ epitope that could bind to HLA-A*02:01 (Mishto et al. 2019). Future studies will need to determine how common and uniform this event is across different cancer cells.

Taken together, there is now ample evidence that mKRAS is sufficiently immunogenic in the context of common HLA class I alleles to elicit T cell responses. Exciting proof-of-concept clinical data with KRAS-specific TILs and TCRs affirm mKRAS as a valid immunotherapy target and support further preclinical development and translation of high-affinity TCRs targeting mKRAS.

## MUTANT p53–TARGETED T CELL THERAPY

### p53 Function in Normal and Cancer Cells

*TP53* (tumor protein 53) is the most commonly mutated gene in human cancers, with aberrations detected in over 50% of cases across all malignancies. p53 is a transcription factor, and under normal conditions, its stability is tightly regulated by the ubiquitin ligase MDM2 (Tang et al. 2020). DNA damage and cellular stress promote p53 phosphorylation and acetylation, which can enhance p53 function and prevent its degradation. The higher levels of activated p53 can then induce cell cycle arrest until the DNA damage is repaired or stressors are neutralized. Alternatively, when DNA damage is too excessive, p53 kick-starts programmed cell death. Because of its critical role in preserving genome integrity and preventing the propagation of error-riddled DNA, p53 has been dubbed the guardian of the genome (Tang et al. 2020).

Most p53 mutations involve exons 5–8, which participate in DNA binding and lead to a de facto loss of p53 function. In the absence of functional p53, cancer cells proliferate, accumulate additional genomic changes, and resist apoptosis in the face of stress and nutrient deprivation. Based on the observations that p53^wt/null^ and p53^wt/mutant^ mice exhibit different patterns of tumor growth and metastasis, several groups proposed that mutant p53 can antagonize remaining wild-type p53, or that mutant forms of p53 can act as gain-of-function oncogenes that directly promote invasion, metastasis, and chemoresistance (Alexandrova et al. 2015, Boettcher et al. 2019). As is the case with KRAS, most p53 mutations are missense mutations leading to single–amino acid changes. However, unlike KRAS, for which there are only three hotspots for driver mutations, mutations in p53 are more diverse, with the most common ones being changes at codons 175, 220, 245, 248, 249, 273, and 282 (Malekzadeh et al. 2019).

Since the discovery of mutant forms of p53, many groups have attempted to reactivate its normal function. Several compounds that restore proapoptotic function to mutant p53 have been described and are being tested in the clinic (Hu et al. 2021). PC-14586 has been shown to selectively bind to a crevice exclusively found in the Y220C p53 mutant protein and stabilize the protein in the wild-type conformation, thereby promoting DNA binding and expression of p53 target genes. It is currently being evaluated in a phase I/II clinical trial (NCT04585750), and a preliminary report demonstrated an overall response rate of 32% in treated patients across six tumor types (Dumbrava et al. 2022). APR-246 is another candidate compound, which was recently granted a breakthrough therapy designation by the FDA (US Food and Drug Administration) based on its ability to stabilize the R175H p53 mutant in a functional conformation (Sallman et al. 2021).

### Mutant p53 Is Immunogenic

Evidence that mutant p53 could be immunogenic was initially provided by studies in mice (Noguchi et al. 1994). BALB/c mice vaccinated with mutant p53 developed detectable mutant p53–specific CD4^+^ and CD8^+^ T cells with cytotoxic activities. Immunized mice were significantly protected against challenge with the p53-mutant Meth A sarcoma line, suggesting mutant p53 peptides are processed and presented by tumor cells. Subsequent studies showed that both mutant and wild-type p53 can elicit a T cell response in HLA-A*02:01-transgenic and in peripheral blood T cells isolated from patients with cancer (Theobald et al. 1995, Tilkin et al. 1995). Vaccination of patients with mutant p53 peptide-pulsed autologous mononuclear cells led to expansion of reactive T cells and a modest improvement in overall survival, suggesting the potential utility of immunologically targeting mutant p53 (Carbone et al. 2005).

Recent studies at the National Cancer Institute (NCI) have provided additional evidence that mutant p53 can be recognized by patient’s CD4^+^ and CD8^+^ T cells. Deniger et al. (2018) isolated and expanded TILs from nine patients with metastatic ovarian cancer and discovered CD4^+^ T cell reactivity to tumor-intrinsic mutant p53 in two of them. In subsequent studies, TIL responses to hotspot p53 mutations were evaluated in more than 40 patients with different tumor types, and both CD4^+^ and CD8^+^ T cell reactivity was demonstrated in 22 individuals (Kim et al. 2022, Malekzadeh et al. 2019).

### Developing Adoptive T Cell Therapy Targeting Mutant p53

Kim et al. (2022) recently isolated TILs from 12 patients with p53-mutant cancers and identified T cell reactivities to previously described hotspot mutations, as well as additional mutations and insertion/deletions in *TP53*. TILs were expanded and reinfused into patients, with 2 of 12 patients experiencing a partial response that lasted 4–6 months. The frequency of mutant p53–specific T cells in the infusion product varied across the cohort (1–50.8%), and most T cells expressed high levels of inhibitory receptors PD-1, TIM-3, and LAG-3, indicating dysfunction/exhaustion may have interfered with efficacy in these patients.

To overcome the limitations of TIL transfer, researchers at the NCI and from our lab have characterized a panel of TCRs recognizing mutant p53 (Kim et al. 2022; Lo et al. 2019; Malekzadeh et al. 2019, 2020). Of these, TCRs targeting the p53 mutation R175H in the context of HLA-A*02:01 represent particularly high-value reagents that could be used to treat ~9,000 newly diagnosed patients annually in the United States alone (Pearlman et al. 2021). Such R175H-specific TCRs were isolated from TILs and peripheral blood lymphocytes of several patients and recognize target cells transfected with a p53 R175H minigene as well as tumor lines naturally expressing the p53 mutation (Kim et al. 2022; Lo et al. 2019; Malekzadeh et al. 2019, 2020). TCR targeting of R175H:HLA-A*02:01 was further evaluated by Kim et al. (2022), revealing that healthy donor T cells transduced with this TCR can kill tumor cells in NSG mice. The efficacy of this TCR was then evaluated clinically in an HLA-A*02:01^+^ patient whose tumor expressed R175H and had progressed after 10 prior lines of therapy. Peripheral blood T cells from this patient were transduced with the R175H-specific TCRs, expanded in vitro, and reinfused after a standard lymphodepleting regimen. She experienced significant acute toxicity, including grade 3 cytokine release syndrome, requiring treatment with vasopressors and steroids. At 6 and 14 weeks after treatment, there was a significant decrease in target lesions (by 37% and 55%, respectively). However, this patient progressed at 6 months, and succumbed to disease at 8 months, after treatment start. Tumor biopsy revealed a retained expression of mutant p53 and the presence of TCR-transduced T cells, but whole-exome sequencing revealed loss of heterozygosity at the HLA-A*02:01 locus. This proof-of-concept study, while identifying obstacles, supports the further development of mutant p53–targeted TCR-based therapies and warrants a deeper dive into mechanisms of TCR therapy resistance.

## ADOPTIVE T CELL THERAPY TARGETING VIRAL ONCOPROTEINS

An estimated 10–12% of cancers are associated with or are directly caused by viral infections, with over 80% occurring in the developing world (Mesri et al. 2014). Infections with human papillomavirus (HPV) and Epstein-Barr virus (EBV) account for over 800,000 new cancer diagnoses annually and span a variety of histologies, including HPV-associated cervical, vaginal, and head and neck cancers, as well as EBV-associated lymphoma, nasopharyngeal carcinoma, and gastric cancers (Smith & Khanna 2017, Sun et al. 2020). Individuals coinfected with HIV or immunosuppressed from having undergone a transplant procedure are particularly vulnerable to HPV- and EBV-mediated oncogenesis (Bushara et al. 2022, Smith & Khanna 2017).

Virus-driven oncogenesis is a complex process that requires viral genome integration or maintenance in infected cells and expression of viral oncoproteins. These viral oncoproteins initiate a cascade of events that ultimately subvert host cell cycle regulation, allow for DNA damage to go unchecked, and promote immune evasion. For example, a subset of mucosal cells infected with HPV serotype 16 or 18 maintain expression of the E6 and E7 viral oncoproteins, which directly inhibit tumor suppressors p53 and pRb, pushing the infected cell into the S phase of the cell cycle. This promotes sustained cell proliferation concomitant with genomic instability and can result in DNA replication errors with accrual of oncogenic mutations. In addition, E6 and E7 also dampen immune responses by downregulating HLA class I expression on infected cells (Mesri et al. 2014).

Prophylactic vaccines targeting HPV strains 16 and 18 have contributed to a global reduction in HPV-associated tumor burden (Drolet et al. 2015). However, such vaccines are ineffective in patients with precancerous lesions or established cancers, in part because they do not include antigens for priming responses against E6 and E7 (Smith & Khanna 2017). Therapeutic vaccines targeting E6 and E7 have been effective in eliminating premalignant vulvar and cervical lesions, but further development is required to achieve efficacy in advanced cancers (Kenter et al. 2009, Trimble et al. 2015). As adoptive transfer of virus-specific T cells can restore antiviral immunity (Riddell et al. 1992) and virus-driven tumors stably express viral oncoproteins as noted above, there has been a push to harness endogenous E6/E7-specific T cells or redirect T cell specificity to the neoantigens encoded by these viral proteins.

HPV-specific CD8^+^ T cells have been detected in patients with cervical cancer, and transfer of ex vivo expanded E6/E7-specific TILs led to objective responses in three of nine patients from a 2015 study (Stevanovic et al. 2015). HPV-specific TCRs targeting E6 or E7 in the context of HLA-A*02:01 have been isolated and tested in phase I/II clinical trials with encouraging results (Doran et al. 2019, Nagarsheth et al. 2021). In total, 2 of 12 patients treated with autologous CD8^+^ T cells transduced with an E6:HLA-A*02:01-specific TCR experienced an objective response (Doran et al. 2019), and 6 of 12 patients benefitted from therapy with an E7:HLA-A*02:01-targeted TCR (Nagarsheth et al. 2021). Epitope processing and presentation, TCR functional avidity, and infusion product cell phenotypes are all factors that could contribute to the observed differences in objective responses.

EBV-targeted ACT was pioneered for the treatment of hematopoietic stem cell transplant patients who developed EBV-driven posttransplant lymphoproliferative disease (PTLD). Treatment with adoptively transferred donor-derived T cells, expanded in vitro in the presence of EBV-transformed donor-derived B cells, led to durable responses in transplant patients, with transferred T cells persisting up to 9 years postinfusion (Heslop et al. 1996, 2010; Rooney et al. 1998). In PTLD, EBV genomes are maintained as episomes in infected cells, and all EBV viral proteins are expressed and potentially targetable. However, in EBV-driven lymphomas and carcinomas, the viral genome has integrated, and the transformed cells express a limited subset of EBV latency proteins including EBNA1, LMP1, and LMP2 that promote RAG1/2 reactivation, B cell proliferation, and immune evasion (Munz 2020). To provide de novo EBV-targeted immune responses in these patients, several groups turned to developing TCR-based therapies. In fact, EBV is now one of the major targets in cell therapy trials for solid tumors (Upadhaya et al. 2021). The EBV-specific TCR toolbox includes TCRs specific for LMP1:HLA-A*02:01, LMP2:HLA-A*02:01, and EBNA3B:HLA-A*11:01, among others (Cho et al. 2018, Orentas et al. 2001, Schaft et al. 2006). Lessons learned from EBV-TCR clinical trials will undoubtedly inform future TCR therapy design for other virus-driven cancers.

Merkel cell polyomavirus (MCPyV) is the most recently discovered oncovirus, and the causative agent of ~80% of Merkel cell carcinoma (MCC) cases (Feng et al. 2008, Spurgeon & Lambert 2013). MCPyV is present in and chronically shed from the skin of most individuals. However, in rare individuals, environmental factors, aging, or immunosuppression can lead to MCPyV genome integration into a subset of skin cells, which can then lead to constitutive expression of large and small T antigen oncoproteins and result in MCC (Spurgeon & Lambert 2013). MCPyV T antigens inhibit the pRb tumor suppressor, promote cell cycle progression, and are required for tumor cell survival and malignancy (Spurgeon & Lambert 2013). MCPyV T antigen–specific CD4^+^ and CD8^+^ T cells can be detected in MCC patients’ tumors and peripheral blood but are largely dysfunctional (Afanasiev et al. 2013, Iyer et al. 2011, Jing et al. 2020). At our institution, adoptive transfer of ex vivo–expanded polyclonal MCPyV large T antigen–specific T cells led to a durable response in a patient with metastatic MCC, with functional T cells persisting for more than 200 days (Chapuis et al. 2014). These provocative results prompted the launch of a phase I/II clinical trial testing the efficacy of MCPyV T antigen–specific T cells restricted to HLA-A*02:01 in patients with unresectable or metastatic MCC (NCT03747484). In five patients treated to date, transfer of MCPyV-specific autologous TCR-transduced CD8^+^ T cells proved safe, and transduced T cells were detectable in the tumor lesions for over one month after treatment (Veatch et al. 2022c). However, evasion by downregulation of HLA class I has been noted, and the addition of therapies that enhance class I expression preceding MCPyV-TCR T cell transfer is being explored (Veatch et al. 2022a).

## EXISTING CHALLENGES AND FUTURE DIRECTIONS

TCR-based therapies directed against public neoantigens have already shown promise in the clinic but will require further improvement to become reproducibly effective. Factors that can limit TCR T cell efficacy include suboptimal TCR avidity for the target neoantigen, variable TCR expression, progressive T cell loss, dysfunction of persisting cells in response to chronic stimulation or an immunosuppressive TME, and emergence of HLA-low or HLA-negative tumor cells. As briefly described below, there has been significant recent progress toward overcoming these obstacles and building next-generation TCR T cells.

TCR avidity for cognate antigen directly impacts T cell function, as neoantigen-specific T cells with a low avidity exhibit poor tumor control, while high-avidity T cells can be rapidly driven to exhaustion or clonal deletion (Pai et al. 2019, Shakiba et al. 2022). Therefore, defining the optimal avidity range of neoantigen-specific TCRs to provide durable clinical benefit, and the factors that influence that range, remains a complicated issue, particularly because target epitope:MHC expression can be heterogeneous within the tumor. TCR expression levels strongly influence functional avidity and T cell fate in the TME; thus, achieving stable, predictable, and physiologically relevant TCR expression has been a major engineering goal (Shakiba et al. 2022). Current platforms generally rely on transduction of TCRs with retroviral or lentiviral vectors, but this approach does not eliminate the endogenous TCR α/β chains that can directly compete for the limiting signaling machinery components required for TCR surface expression or mispair with the therapeutic TCR chains, an event that can both reduce expression of the desired TCR complex and create potentially dangerous autoreactive TCRs. Modifications to the introduced TCR chains, such as codon optimization to enhance translation and insertion of cysteine mutations to create a disulfide bond between the chains to promote appropriate pairing, facilitate assembly in the endoplasmic reticulum, and improve competition for TCR complex components and export to the cell surface, have partially addressed the problem (Kuball et al. 2009). More recently, CRISPR/Cas9 gene-editing reagents have provided a more complete solution, making it possible to knock out the endogenous *TCRA*, or both *TCRA* and *TCRB*, and provide the therapeutic TCR either through a viral vector or as a homology repair template into the *TCRA* locus (Roth et al. 2018; Schober et al. 2019, 2020). Orthotopic TCR replacement in the *TCRA* locus has yielded near-physiological control of TCR expression, and evolving strategies that enhance the efficiency and safety of this gene-editing strategy should lead to broader adoption of this approach in clinical trials.

**Figure 1.**
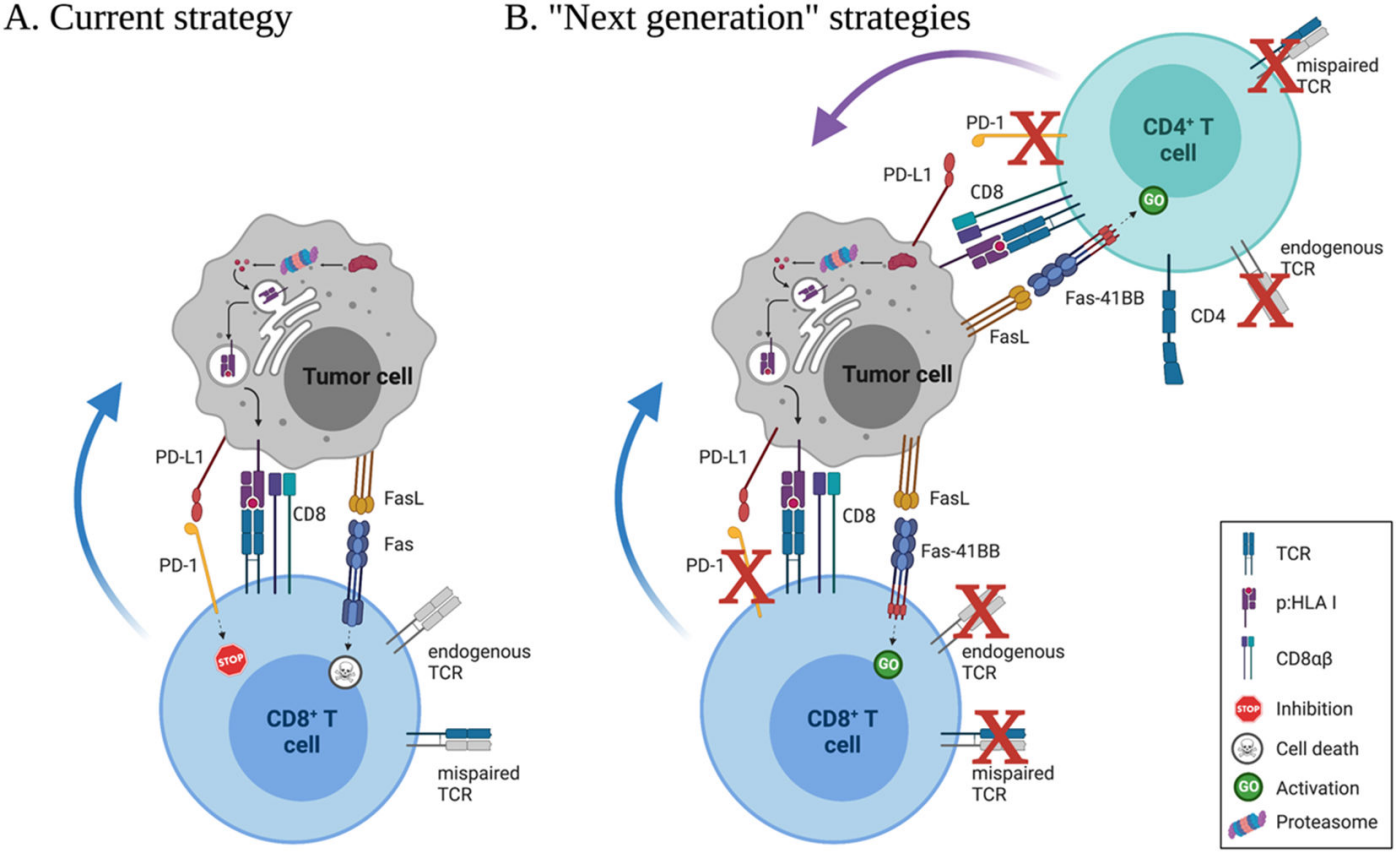
Next generation strategies for TCR-based adoptive T cell therapies. (A) Current TCR-engineering strategies include retrovirus- or lentivirus-mediated delivery of the therapeutic TCR to CD8^+^ T cells. These TCR-transduced cells retain the endogenous TCR, and may express mispaired receptors on their surface. TAA or TSA are generated in the tumor cells through proteosome-mediated protein degradation in the cytosol, and are loaded onto HLA class I in the endoplasmic reticulum. In the TME, TCR-engineered T cells interact with peptide:HLA class I complexes presented on tumor cells, and deliver cytotoxic cargo leading to tumor cell death. TCR-engineered T cells eventually lose their cytotoxic function through a variety of mechanisms, including inhibitory signaling through PD-1 and death-signaling through Fas. (B) Emerging strategies involve CRISPR/Cas9-facilitated orthotopic replacement of the TCR (to promote physiological expression of the therapeutic receptor and eliminate the endogenous TCR), inhibitory receptor genetic deletion (to prevent inhibitory signaling), expression of immunomodulatory fusion proteins (such as Fas/4-1BB, to convert a death or inhibitory signal into an activation signal), and/or CD4^+^ T cell engineering (with addition of the therapeutic TCR HLA class I-restricted TCR, CD8αβ, and immunomodulatory fusion proteins). Created with Biorender.com.

Evidence from TAA-targeted TCR therapy suggests that inserting the TCR alone into CD8^+^ T cells may not be enough to mediate durable responses. In solid tumors, T cells encounter immunosuppressive cells and factors, nutrient deprivation, and chronic antigen stimulation (Anderson et al. 2017). Recent developments in genetic engineering and synthetic biology have prompted the design and implementation of numerous strategies that could counter these obstacles and enhance CD8^+^ T cell survival and function. For instance, recently we and others armed CD8^+^ T cells with immunomodulatory fusion proteins (e.g., CD200R/CD28, PD-1/CD28, or Fas/4–1BB) or chimeric cytokine receptors (e.g., IL-4/IL-7) that can convert inhibitory/death signals into activating and survival signals that enhance T cell function and persistence (Anderson et al. 2022; Ankri et al. 2013; Leen et al. 2014; Oda et al. 2017, 2020). Several groups genetically disrupted inhibitory receptor signaling or used ICB after T cell transfer, while others perturbed epigenetic programs that commonly drive progressive T cell dysfunction (John et al. 2013, Prinzing et al. 2021, Ren et al. 2017). How best to incorporate any or all of these strategies to create maximally effective TCR T cell products for distinct tumor therapy settings remains to be determined, but the potential for synthetic biology and genetic engineering to improve therapeutic efficacy seems unequivocal (Figure 1).

Most TCR T cell therapies have focused on engineering CD8^+^ T cells, but CD4^+^ T cells have been shown to promote CD8^+^ T cell function and expansion and to delay or prevent CD8^+^ T cell exhaustion (Alspach et al. 2019). Tumor-specific CD4^+^ T cells, particularly those targeting neoantigens, can also mediate tumor regression in mice and in patients, either directly by killing tumor cells or indirectly by secreting cytokines and chemokines that collectively reprogram myeloid cells in the TME and enhance CD8^+^ T cell function (Greenberg et al. 1981, 1985; Hunder et al. 2008; Tran et al. 2014; Veatch et al. 2018, 2019, 2022b). As most nonhematopoietic tumors do not express HLA class II, we and others have engineered CD4^+^ T cells to recognize and respond to epitopes presented in the context of HLA class I by providing both an HLA class I–restricted TCR and the CD8αβ coreceptor (Lahman et al. 2022, Rath et al. 2020). Dually transduced CD4^+^ T cells demonstrated specific and potent cytotoxicity, as well as the ability to provide help to CD8^+^ T cells during chronic antigen challenge (Lahman et al. 2022, Rath et al. 2020). Future clinical studies will determine if infusion of both CD4^+^ T cells and CD8^+^ T cells targeting the same antigen can improve effector cell function and persistence in vivo, delay/prevent exhaustion, and obviate the need for either repeated in vivo IL-2 injections or vaccine-based boosting (Hanada et al. 2019, Stromnes et al. 2015).

A major obstacle for TCR T cell therapies is the genetic loss or, more commonly, reduced transcription of HLA class I genes. Permanent genetic loss of the restricting HLA allele has been noted after adoptive transfer of mKRAS-specific TILs, mutant-p53-specific TCR T cells, or HPV-specific TCR T cells, and it may require concurrent or sequential targeting of multiple different peptide:HLA complexes (Doran et al. 2019, Kim et al. 2022, Tran et al. 2016). By contrast, transcriptional HLA reduction/loss, which is likely the more common mechanism, does appear to be reversible, as tumor cells harvested at the time of progression after MCPyV-T cell therapy can be induced to reexpress HLA class I after in vitro treatment with interferon-γ or exposure to 5-azacitidine, a hypomethylating agent (Paulson et al. 2018). A genome-wide screen of HLA class I–low tumor cells also revealed that polycomb repressor complex 2 (PRC2) can silence HLA expression, and that in vitro PRC2 inhibition with drugs such as EZH2 inhibitors can enhance HLA expression (Burr et al. 2019). Strategies to efficiently determine the operative mechanism of HLA class I reduction in patients’ tumor cells are needed, and studies in preclinical models should prove valuable for determining the optimal dosing and timing relative to TCR T cell transfer of agents such as the ones noted above.

## CONCLUSIONS

In summary, public neoantigens, particularly those associated with driver oncogenes, represent an elite class of tumor rejection antigens, as they are (*a*) present in all cells within a tumor, (*b*) absent from normal cells and tissues, (*c*) required for tumor growth or survival, and (*d*) shared across patients. Although a limited number of such public neoantigens have been validated as immunogenic, the evidence discussed in this review strongly supports the hypothesis that these neoantigens are actionable and targetable with TCR T cell–based therapies or, as recently described, with bispecific antibodies that contain two binding domains, one derived from a TCR-mimic antibody highly specific for a distinct peptide/MHC complex that can target the tumor, and a second for CD3 that can capture and activate proximal T cells (Douglass et al. 2021, Hsiue et al. 2021). Exciting times are ahead, as advances in genome engineering, synthetic biology, and epigenetic modulation will allow for the rational addition of approaches to enhance T cell potency or circumvent mechanisms of resistance to therapy.

## Figures and Tables

**Table 1.** Validated immunogenic public neoantigens.

Type	Neoantigen	HLA restriction	Antigen validation	TCR isolation/validation
**Missense mutations**	CDK4 R24C	A*02:01	(Lennerz et al 2005, Wolfel et al 1995)	(Leisegang et al 2016)
	CREBBP R1446C	B*07:02	(Nielsen et al 2016)	NA
	KRAS G12V	A*03:01, A*11:01, A*30:01, A*68:01, C*01:02, C*03:03, C*03:04	(Bear et al 2021, Choi et al 2021, Martinov et al 2022, Wang et al 2016)	(Bear et al 2021, Choi et al 2021, Martinov et al 2022, Wang et al 2016)
	KRAS G12D	A*03:01, A*11:01, A*68:01, B*07:02, C*03:04, C*08:02	(Bear et al 2021, Choi et al 2021, Martinov et al 2022, Tran et al 2016, Wang et al 2016)	(Bear et al 2021, Choi et al 2021, Leidner et al 2022, Martinov et al 2022, Tran et al 2016, Wang et al 2016)
	P53 R175H	A*02:1	(Deniger et al 2018, Malekzadeh et al 2019)	(Deniger et al 2018, Kim et al 2022, Malekzadeh et al 2019)
	BRAF V600E	A*02:01, B*27:05	(Andersen et al 2004, Veatch et al 2018)	(Andersen et al 2004, Veatch et al 2018)
	IDH1 R132H	DRA*0101/DRB1*0101	(Platten et al 2021, Schumacher et al 2014)	(Platten et al 2021)
	IDH2 R140Q	B*07	(Wang et al 2019)	NA
	PIK3CA H1047L	A*03:01	(Chandran et al 2022)	(Chandran et al 2022)
**Frameshift insertions/deletions**	NPM1	A*02:01	(van der Lee et al 2019)	(van der Lee et al 2019)
	TGFβR2	A*02:01	(Inderberg et al 2017)	(Inderberg et al 2017)
**Gene translocations**	BCR-ABL	A*03:01	(Norbury et al 2000)	NA
	CBFB-MYH11	B*40:01	(Biernacki et al 2020)	(Biernacki et al 2020)
**Viral oncoproteins**	HPV E6	A*02:01	(Kenter et al 2009)	(Doran et al 2019)
	HPV E7	A*02:01	(Trimble et al 2015)	(Nagarsheth et al 2021)
	EBV EBNA1	B*7, B*35:01, B*53	(Blake et al 2000)	NA
	EBV EBNA3A	B*08:02	(Schaft et al 2006)	(Schaft et al 2006)
	EBV LMP1	A*02:01	(Cho et al 2018, Sing et al 1997)	(Cho et al 2018)
	EBV LMP2	A*02:01, A*11:01	(Orentas et al 2001, Sing et al 1997, Zheng et al 2015)	(Orentas et al 2001, Zheng et al 2015)
	MCPyV LT-Ag	A*02:01	(Chapuis et al 2014, Paulson et al 2018)	(Gavvovidis et al 2018)
